# Supplementary figures and images for: Divergence of cochlear transcriptomics between reference‑based and reference‑free transcriptome analyses among Rhinolophus ferrumequinum populations
Source: PLoS One. 2023 Jul 11;18(7):e0288404. doi: 10.1371/journal.pone.0288404 (PMC10335675; doi:10.1371/journal.pone.0288404)

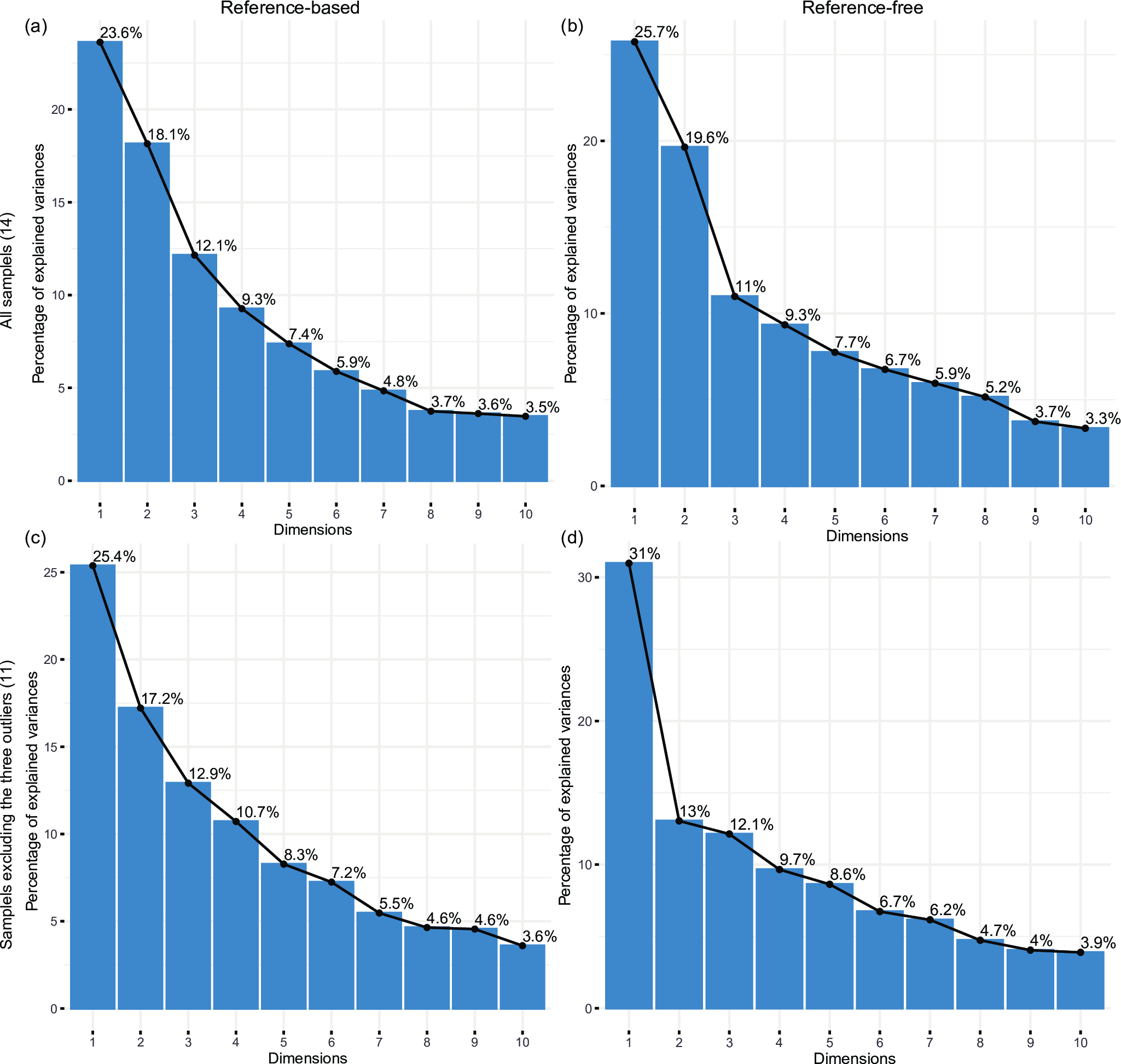

Supplement: S1 Fig — Scree plot of all 14 individuals based on the reference-based method (a) and the reference-free method (b). Scree plot of 11 individuals excluding three outlier samples based on the reference-based method (c) and the reference-free method (d). (TIF) [file pone.0288404.s026.tif]

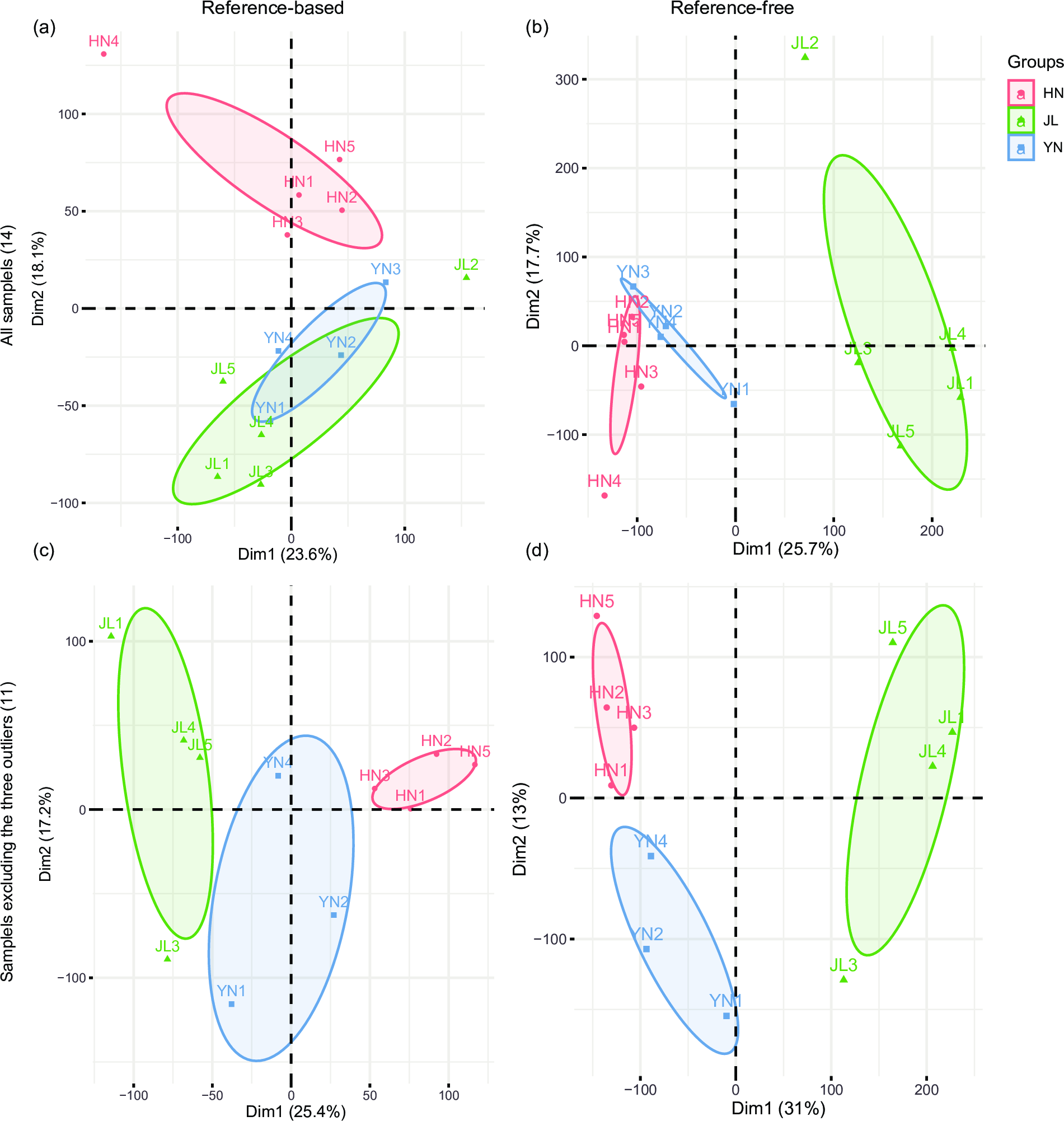

Supplement: S2 Fig — PCA plot of all 14 individuals based on the reference-based method (a) and the reference-free method (b). PCA plot of 11 individuals excluding three outlier samples based on the reference-based method (c) and the reference-free method (d). (TIF) [file pone.0288404.s027.tif]

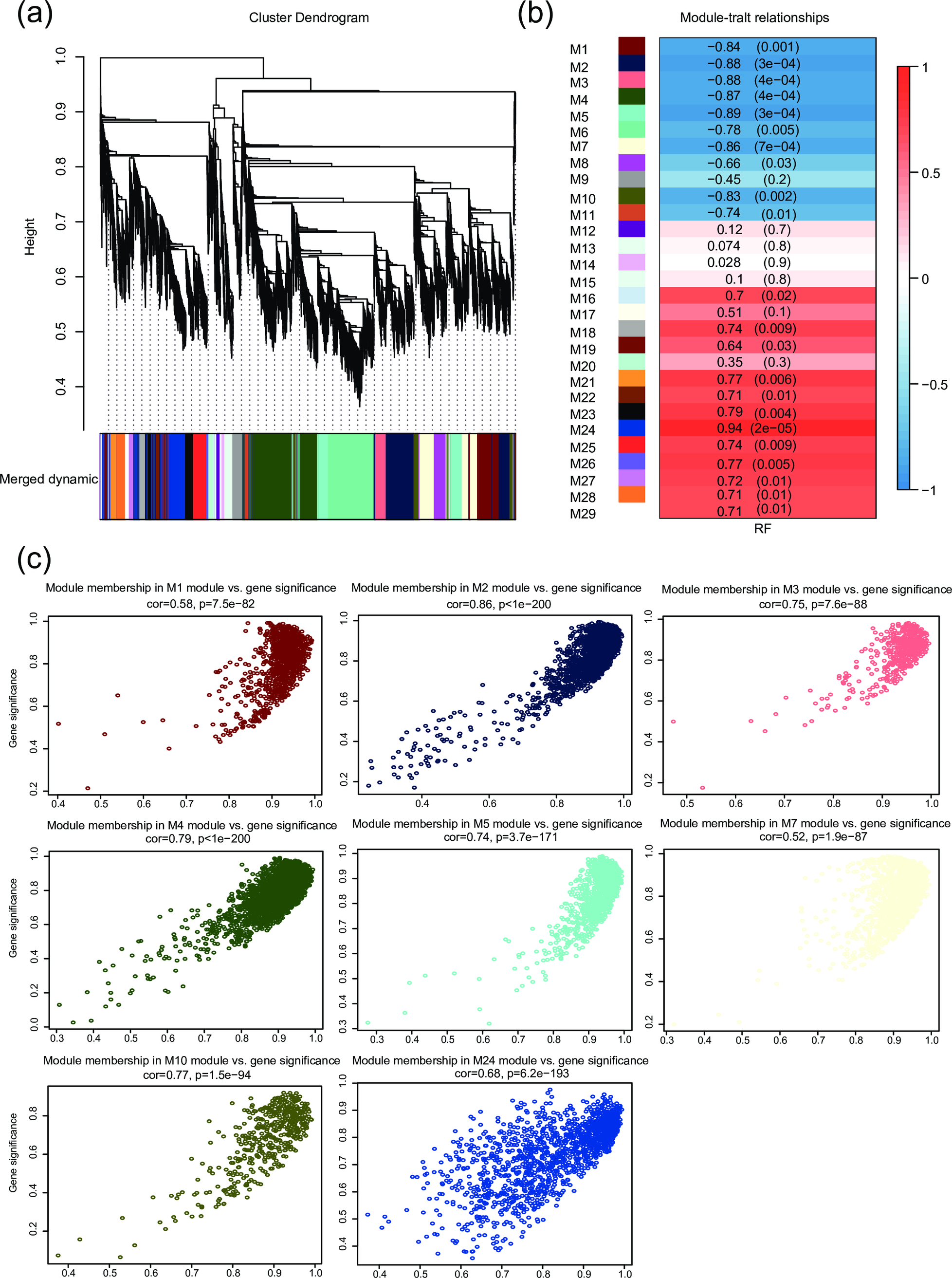

Supplement: S3 Fig — (a) Gene tree spectrum obtained by average linkage hierarchical clustering. (b) Table of module–trait relationships. The correlation coefficient values between the modules and RF phenotype are plotted at the top of each module-trait relationship squares. The p-values were labeled under the correlation coefficients in parentheses. (c) Scatter plots showing module membership and gene significance of genes in modules significantly associated with RF phenotypes. (TIF) [file pone.0288404.s028.tif]

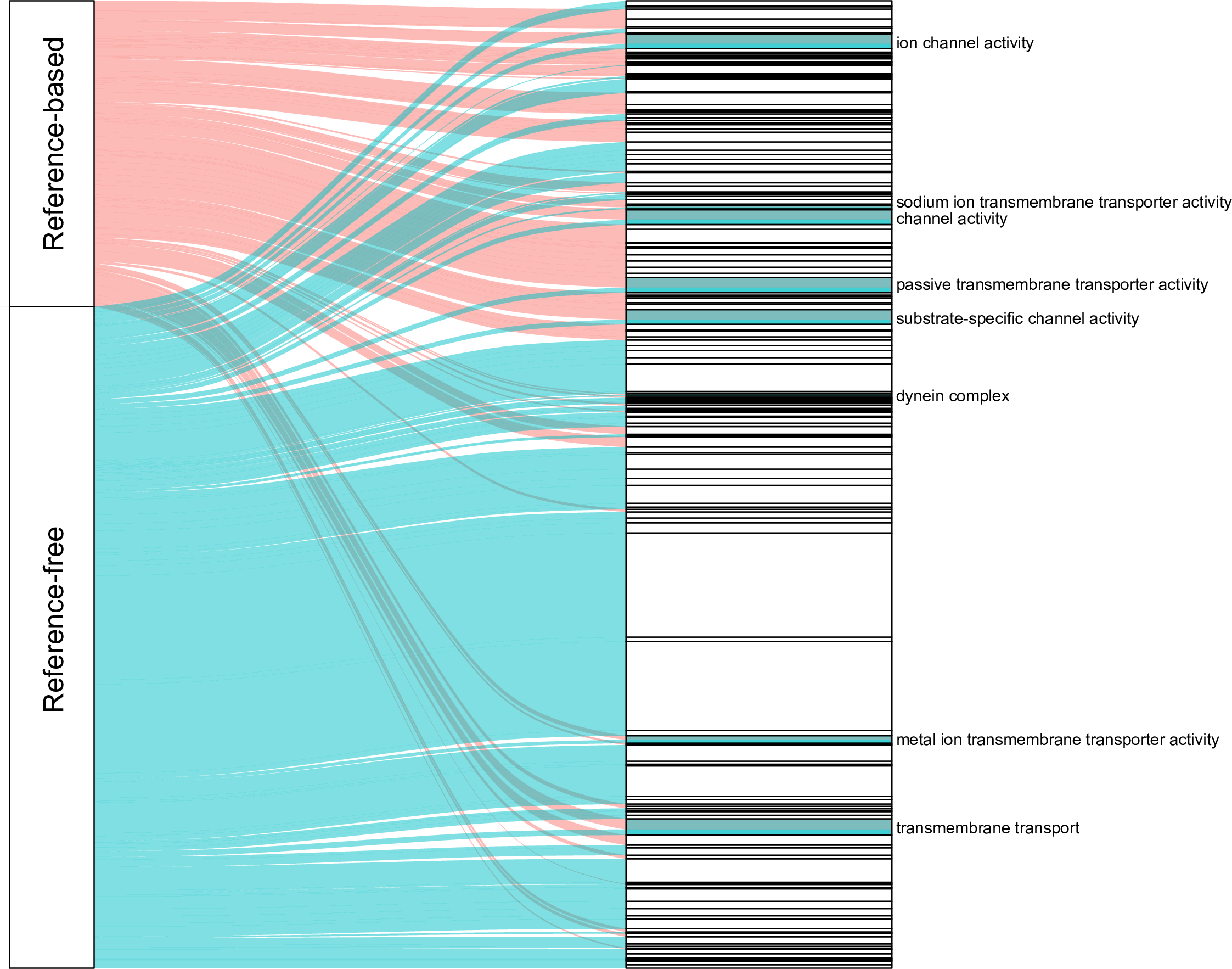

Supplement: S4 Fig — Terms obtained by both methods were colored. The width of the rectangle shape represents the number of gene counts enriched in the terms. (TIF) [file pone.0288404.s029.tif]
