# Supplementary material for: Divergence of cochlear transcriptomics between reference‑based and reference‑free transcriptome analyses among Rhinolophus ferrumequinum populations
Source: PLoS One. 2023 Jul 11;18(7):e0288404. doi: 10.1371/journal.pone.0288404 (PMC10335675; doi:10.1371/journal.pone.0288404)
Supplement: Attachment — Submitted filename: Response to Reviewers.docx [file pone.0288404.s030.docx]

**All of blue sections are our responses to the comments from editors and reviewers**

Editors’ Comments to Author:

Thank you for submitting your manuscript to PLOS ONE. After careful consideration, we feel that it has merit but does not fully meet PLOS ONE’s publication criteria as it currently stands. Therefore, we invite you to submit a revised version of the manuscript that addresses the points raised during the review process.

Response: We are very appreciative of the constructive comments on the manuscript from the reviewers. We have carefully and seriously revised the manuscript according to the comments. These comments are very helpful for us to revise and improve our manuscript. For each comment, our responses are as follows:

Journal Requirements:

When submitting your revision, we need you to address these additional requirements.

1. Please ensure that your manuscript meets PLOS ONE's style requirements, including those for file naming. The PLOS ONE style templates can be found at

https://journals.plos.org/plosone/s/file?id=wjVg/PLOSOne_formatting_sample_main_body.pdf and https://journals.plos.org/plosone/s/file?id=ba62/PLOSOne_formatting_sample_title_authors_affiliations.pdf

Response: We changed the following parts in the manuscript to meet PLOS ONE's style requirements.

1. We adjusted the format of the first page, and added lacking Author Byline (please see page 1).

2. We standardized the format of Table 1 (please see page 11 and 12).

3. We added page number under each page.

4. We deleted all unpublished contents in the reference.

5. We renamed all files as required.

2. We note that the grant information you provided in the ‘Funding Information’ and ‘Financial Disclosure’ sections do not match.

When you resubmit, please ensure that you provide the correct grant numbers for the awards you received for your study in the ‘Funding Information’ section.

Response: We have checked the grant numbers for the awards when we resubmit the manuscript.

3. Thank you for stating the following financial disclosure:

"This work was supported by the National Natural Science Foundation of China (Grant Nos. 32171525 and 31961123001) and Jilin Provincial Natural Science Foundation (Grant No. 20220101291JC)."

Please state what role the funders took in the study. If the funders had no role, please state: "The funders had no role in study design, data collection and analysis, decision to publish, or preparation of the manuscript."

If this statement is not correct you must amend it as needed.

Please include this amended Role of Funder statement in your cover letter; we will change the online submission form on your behalf.

Response: We ask for changing the financial disclosure to the following statement.

This work was supported by grant nos. 32171525 and 31961123001 from the National Natural Science Foundation of China (to KS and JF), and grant 20220101291JC from the Natural Science Foundation of Jilin Province (to KS). The funders had no role in study design, data collection and analysis, decision to publish, or preparation of the manuscript.

We have included this statement in the new cover letter.

4. Thank you for stating the following in your Competing Interests section:

"The authors declare that there is no conflict of interest."

Please complete your Competing Interests on the online submission form to state any Competing Interests. If you have no competing interests, please state "The authors have declared that no competing interests exist.", as detailed online in our guide for authors at http://journals.plos.org/plosone/s/submit-now

This information should be included in your cover letter; we will change the online submission form on your behalf.

Response: We have included the statement “The authors have declared that no competing interests exist.” in the new cover letter.

5. We noted in your submission details that a portion of your manuscript may have been presented or published elsewhere. [Although we used some reference - free transcriptome data which had been published, our study focused on describing the differences between the results of two different methods. What's more, we had got the permission from the authors of the data.] Please clarify whether this [conference proceeding or publication] was peer-reviewed and formally published. If this work was previously peer-reviewed and published, in the cover letter please provide the reason that this work does not constitute dual publication and should be included in the current manuscript.

Response: We clarify that the original data we used and results of reference-free method described in the manuscript has been published under peer review, but we have quoted and integrated this part with our new reference-base analysis results to obtain the difference between the results of two methods, so it does not constitute a duplicate publication.

We have included this statement in the new cover letter.

6. In your Data Availability statement, you have not specified where the minimal data set underlying the results described in your manuscript can be found. PLOS defines a study's minimal data set as the underlying data used to reach the conclusions drawn in the manuscript and any additional data required to replicate the reported study findings in their entirety. All PLOS journals require that the minimal data set be made fully available. For more information about our data policy, please see http://journals.plos.org/plosone/s/data-availability.

"Upon re-submitting your revised manuscript, please upload your study’s minimal underlying data set as either Supporting Information files or to a stable, public repository and include the relevant URLs, DOIs, or accession numbers within your revised cover letter. For a list of acceptable repositories, please see http://journals.plos.org/plosone/s/data-availability#loc-recommended-repositories. Any potentially identifying patient information must be fully anonymized.

Important: If there are ethical or legal restrictions to sharing your data publicly, please explain these restrictions in detail. Please see our guidelines for more information on what we consider unacceptable restrictions to publicly sharing data: http://journals.plos.org/plosone/s/data-availability#loc-unacceptable-data-access-restrictions. Note that it is not acceptable for the authors to be the sole named individuals responsible for ensuring data access.

We will update your Data Availability statement to reflect the information you provide in your cover letter.

Response: We have added all underlying data to the supporting information of the manuscript (please see page 31 and 33 in the revised manuscript).

7. PLOS requires an ORCID iD for the corresponding author in Editorial Manager on papers submitted after December 6th, 2016. Please ensure that you have an ORCID iD and that it is validated in Editorial Manager. To do this, go to ‘Update my Information’ (in the upper left-hand corner of the main menu), and click on the Fetch/Validate link next to the ORCID field. This will take you to the ORCID site and allow you to create a new iD or authenticate a pre-existing iD in Editorial Manager. Please see the following video for instructions on linking an ORCID iD to your Editorial Manager account: <https://www.youtube.com/watch?v=_xcclfuvtxQ>

Response:	We had added the ORCID iD of the corresponding author.

8. Please upload a copy of Supporting Information Table. S2-S11 Table which you refer to in your text on page 26.

Response: Because we changed several parts in the manuscript, the contents of Supporting Information had been changed. Now we reuploaded all Supporting Information Tables.

**Reviewer: 1**

Reviewers' comments to author:

In the manuscript "Divergence of cochlear transcriptomics between reference‐ based and reference‐ free transcriptome analyses among Rhinolophus ferrumequinum populations", the authors used public reference genome and public RNA-seq dataset of a bat species to compare the performance of reference-based and reference-free methods of RNA-seq data analysis. While I think an in-depth comparison of the two RNA-seq data analysis strategies are definitely worth examining, I also feel that the manuscript appears a bit superficial regarding such comparison in its current status. For example, the authors mentioned that the idea that reference assembly and annotation quality as well as the population-wide polymorphism may affect the performance of the reference-based method but they did not quantitatively test and define such influences using their datasets. I think the manuscript could be significantly improved with such strict technical characterization. The current manuscript reads too descriptive to me especially consider the fact that the RNA-seq data is published data and therefore a general description of the DEG results based on such data might not be innovative enough on its own. Also, the language of this manuscript needs to be substantially improved.

Response: Many thanks for those constructive suggestion, and we fully agree with your comments. According to your comments, we have made the following changes:

1. We have deleted or refined those long-winded and superficial descriptions. And to make the results more qualified, we added several analyses.

We first mapped all unigenes (genes spliced in the reference-free research) to the reference genome using BLASTn v2.11.0 to identify gene sequence locations to show the mapping position on reference genome and splice accuracy of these spliced genes [1]. We also added the difference statistics of the shared DEGs’ recognized sequence length and gene expression levels obtained by two methods. For the result and discussion of these newly added part, we have revised as follows:

“We first mapped all unigenes (genes spliced in the reference-free research) to the reference genome using BLASTn v2.11.0 to identify gene sequence locations [28], and the E-value was set to 1E-5. Locations with the longest mapping length were considered the gene locations.” (lines 135-139, page 8 in the “Materials and methods” section).

“And for DEGs obtained by both methods, we calculated the Spearman correlation coefficient of shared DEGs’ expression levels obtained by the two methods using the stats v4.0.3 R package. We also performed paired Mann–Whitney U test to compare the gene expression level and gene length of each DEG using the rstatix v0.7.1 R package. Gene expression levels of shared DEGs were represented by lg-(FPKM+1) and lg-(RPKM+1) for reference-based and reference-free methods, respectively.” (lines 140-145, page 8 in the “Materials and methods” section).

“Additionally, although almost all unigenes (70275 out of 70704 unigenes) were successfully matched to the reference genome, the identified genes and the results of subsequent analyses were different from the results of reference-based method. The mapping result of unigenes assembled by the reference‑free method showed that several short unigenes should be identified as one gene (S2 and S8 Tables). Lengths of the shared DEGs sequences obtained by the reference‑free method were also significantly shorter than those obtained by the reference‑based method (Fig 2C, S7 Table).” (lines 244-251, page 14 in the “Results and discussion” section).

“Our results showed that although gene expression levels of shared DEGs obtained by the two methods were strongly correlated (Fig 2D), significant expression level differences of the same genes between the two methods were shown by paired Mann–Whitney U test (Fig 2C, S7 Table).” (lines 257-260, page 14-15 in the “Results and discussion” section).

2. As we focus on the difference of results in subsequent transcriptome analyses, it’s hard for us to compare two methods in technical level. So we quoted articles focus on this field and disgusted using their research results.

The idea that the results obtained by reference‑based method could be affected by the accuracy and completeness of the reference genome were quoted from several researches. So, we stated the following sentence in the manuscript.

“Results obtained by the reference‑based method could be affected by the accuracy and completeness of the reference genome [6, 9, 10].” (lines 63-64, page 4 in the “Materials and methods” section).

And the idea that the population-wide polymorphism may affect the performance of the reference-based method were also quoted from several previous researches. So, we stated the following sentence in the manuscript.

“Variations of gene-expression patterns among individuals are missed when using only one single reference genome [6]. Because consensus splice site dinucleotide motifs are used to map reads across splice junctions, genomic variants in the splice site prevents the reads from being mapped to the reference genome, which could result in the incomplete information acquisition [11]” (lines 64-69, page 4-5 in the “Materials and methods” section).

3. We have deleted unnecessary descriptions of the reference-free results [6] and refined those long-winded and superficial descriptions in the “Results and discussion” section.

4. We are sorry for the language problem, and the new manuscript has been carefully edited by a professional language editing service to improve the grammar and readability.

page 8 line 27-29: "In this study, we compared the cochlear transcriptome analysis results of Rhinolophus ferrumequinum from three lineages". The authors might want to briefly introduce "Rhinolophus ferrumequinum" with its common name.

Response: Based on your suggestion, we added the common name “greater horseshoe bat” before “*Rhinolophus ferrumequinum*” in the revised manuscript. We changed the sentence to “In this study, we compared the cochlear transcriptome analysis results of greater horseshoe bats (*Rhinolophus ferrumequinum*) from three lineages …” [lines 32-35, page 3].

page 8 line 31-37: The authors might want to consider improve this section to better reflect the difference between the reference-based and reference-free methods.

Response: In order to better reflect the difference between the reference-based and reference-free methods, we rewrote this part as “The results gained by reference-based results had lower false-positive rates and were more accurate because differentially expressed genes among the three populations obtained by this method had higher had greater reliability and a higher annotation rate. Some important differentially expressed genes, such as *TRPC3*, *GJB3*, and *CDH13*, and phenotype-related enrichment terms, including those related to inorganic molecules and proton transmembrane channels, were also obtained only by the reference-based method. However, the reference‑based method might have the limitation of incomplete information acquisition.” [lines 35-42, page 3].

page 9 line 52: "qualified" -> "quantified"?

Response: Based on your suggestion, we have changed “qualified” to “quantified” [line 56, page 4].

page 9 line 58-62: This sentence needs a rephrasing.

Response: We rewrote the sentence to “Results obtained by the reference‑based method could be affected by the accuracy and completeness of the reference genome [6, 9, 10]. Variations of gene-expression patterns among individuals are missed when using only one single reference genome [6]. Because consensus splice site dinucleotide motifs are used to map reads across splice junctions, genomic variants in the splice site prevents the reads from being mapped to the reference genome, which could result in the incomplete information acquisition [11].” [lines 63-69, page 4-5].

page 10 line 82-83: "which provided a good database for transcriptome analysis with reference genome." => "which provided a good reference genome for transcriptome analysis."

Response: Based on your suggestion, we have changed it to “which provided a good reference genome for transcriptome analysis” [lines 88-89, page 6].

page 10 line 95: "Row data" => "Raw data"

Response: Based on your suggestion, we have changed “Row data” to “Raw data” [line 101, page 6].

page 12 line 106: Please provide the name, version, and command of the corresponding software used for contamination filtering and adapter trimming.

Response: Contamination filtering and adapter trimming were performed during the quality control process, and the corresponding software we used for transcriptome quality control was fastp v0.19.7 [2]. The command was “fastp -g -q 20 -u 50 -n 15 -l 150”. “-q 20 -u 50” means reads with low-quality (Q-value ≤20) base accounting for more than 50% of the total read length would be flittered. “-n 15” limits the count of unknown nucleotides (N) under 15% in one read. “- l 150” means reads less than 150 bases would be flittered.

In the revised manuscript, we added the software name, and rewrote the description of the parameters used in quality control.

“To ensure data analysis quality, raw data were filtered and trimmed using fastp v0.19.7 [24]. We removed reads contaminated by adapter, containing more than 15% ploy-N (N means unknown nucleotides) or containing more than 50% low-quality (Qphred ≤ 20) bases.” [lines 110-113, page 7].

page 18 line 220: What did the authors mean by "gene reading errors"?

Response: We tried to describe it because reference‑free RNA-Seq method has limitations such as gene identification bias, low transcriptome coverage and high false positive rates, the use of reference-free method may not help identifying genes precisely. To make the sentences more accurate and understandable, we have changed “gene reading errors” to “incorrect gene identifications” [line 253-254, page 14].

page 25 line 369: What did the authors mean by "obtain more results"?

Response: To make the sentence clearer, we have changed “obtain more results” to “find more possible DEGs that may be distributed in highly differentiated gene regions of the species’ genome that are missed by the reference-based method.” [lines 385-387, page 21].

Reviewer #2: （1）line 123-124 :should be three populations? or rewrite this sentence more accurately.

Response: Sorry for any confusion. Now we have changed it to “We then performed principal component analysis (PCA) of all individuals using the factoextra v1.0.7 R package to identify outlier individuals, and removed the same outliers as in the reference-free research, JL2, HN4 and YN3 [22]. Then we repeated PCA to produce the clustering result of the remnant samples. All subsequent analyses were performed excluding those three outlier samples.” [lines 122-127, page 7].

（2））Figure1:

1.please explain why sample HN4/JL2/YN3 are not included in the gene clustering result?

2.if you excluded these three samples, have you excluded them in the subsequent analysis?

Response: Sorry for any confusion. About your first question, we first performed principal component analysis (PCA) based on reference-base data of all individuals (please see Supporting Information S1), and found there were individual outliers in all samples. As we want to compare the result of two method, the samples used must be the same as the previous research [3]. Therefore, we filtered the same outlier samples as them and also performed PCA analysis to show the clustering result of remnant samples, in order to confirm the correctness of the outlier choice. The remnant samples were well clustered according to the results of PCA. Thus, sample HN4/JL2/YN3 were not included in the gene clustering result and subsequent analyses. And for your second question, we have excluded them in all subsequent analyses.

In order to make it more explicit, we have changed the sentences to “We then performed principal component analysis (PCA) of all individuals using factoextra v1.0.7 R package to identify outlier individuals, and removed the same outliers JL2, HN4 and YN3 as the reference-free research [22], then repeated PCA to show the clustering result of the remnant samples. All subsequent analyses were performed excluding those three outlier samples.” [lines 122-127, page 7].

（3）Figure2: not labeled

2 types of analytical method: reference-based and reference-free

1. How much do the differentially expressed genes obtained by each analytical means differ between different samples of the same species of bats?

2. Comparison of differentially genes between the two analytical means for the same species of bats.

3. Comparison of differentially expressed genes between bats of different species by the same method.

Response: Our study compared the results obtained by two methods using the same samples and analyses.

To make the figure easier to understand, we changed the contents and legends of Figure 2 to “Fig 2. Comparison of DEGs obtained by pairwise comparisons (HN vs. JL, HN vs. YN, and YN vs. JL) using reference-based method and reference-free methods. (A)Venn diagram showing the number of DEGs obtained by reference‑based and reference‑free methods. (B) The annotation results of DEGs obtained by the two methods. The numbers of annotated DEGs that were shared by the two methods, those that were only obtained by one method, and those without annotations are labeled on the histogram plot.” [lines 218-224, page 13].

And based on your suggestion, to further compare the difference of DEGs between two methods, we added the difference statistics of the shared DEGs’ recognized sequence length and gene expression levels obtained by two methods since they were comparable.

For the result and discussion of these newly added part, we have revised as follows:

“And for DEGs obtained by both methods, we calculated the Spearman correlation coefficient of shared DEGs’ expression levels obtained by the two methods using the stats v4.0.3 R package. We also performed paired Mann–Whitney U test to compare the gene expression level and gene length of each DEG using the rstatix v0.7.1 R package. Gene expression levels of shared DEGs were represented by lg-(FPKM+1) and lg-(RPKM+1) for reference-based and reference-free methods, respectively.” (lines 140-145, page 8 in the “Materials and methods” section).

“Lengths of the shared DEGs sequences obtained by the reference‑free method were also significantly shorter than those obtained by the reference‑based method (Fig 2C, S7 Table).” (lines 249-251, page 14 in the “Results and discussion” section).

“Our results showed that although gene expression levels of shared DEGs obtained by the two methods were strongly correlated (Fig 2D), significant expression level differences of the same genes between the two methods were shown by paired Mann–Whitney U test (Fig 2C, S7 Table).” (lines 257-260, page 14-15 in the “Results and discussion” section).

**References:**

[1] McGinnis S, Madden TL. BLAST: at the core of a powerful and diverse set of sequence analysis tools. Nucleic Acids Res. 2004;32(Web Server issue):W20-5. https://doi.org/10.1093/nar/gkh435 PMID: 15215342

[2] Chen S, Zhou Y, Chen Y, Gu J. fastp: an ultra-fast all-in-one FASTQ preprocessor. Bioinformatics. 2018;34(17):i884-i90. 10.1093/bioinformatics/bty560 PMID: 30423086

[3] Zhao H, Wang H, Liu T, Liu S, Jin L, Huang X, et al. Gene expression vs. sequence divergence: comparative transcriptome sequencing among natural Rhinolophus ferrumequinum populations with different acoustic phenotypes. Frontiers in Zoology. 2019;16:37. https://doi.org/10.1186/s12983-019-0336-7 PMID: 31528181
