# Supplementary material for: Divergence of cochlear transcriptomics between reference‑based and reference‑free transcriptome analyses among Rhinolophus ferrumequinum populations
Source: PLoS One. 2023 Jul 11;18(7):e0288404. doi: 10.1371/journal.pone.0288404 (PMC10335675; doi:10.1371/journal.pone.0288404)
Supplement: Attachment — Submitted filename: Response to Reviewers.docx [file pone.0288404.s031.docx]

**All of blue sections are our responses to the comments from reviewers**

**Reviewer: 1**

Reviewers' comments to author:

The revised manuscript has been largely improved in terms of structure and readability. Most of my previous comments have been addressed by the authors. However, if possible, I would like to ask the authors to make additional efforts (see the major concern below) as I feel this is important.

Major concern:

The reference-based method implemented in this study uses FPKM for transcript quantification, whereas the reference-free method implemented in the cited study (Zhao et al. 2019) uses RPKM instead. Therefore, it is difficult to conclude how much of the observed DEG difference is due to the FPKM vs RPKM contrast and how much of it is due to the reference-based vs. reference-free contrast. Therefore, I would recommend the authors to re-run the reference-free analysis with FPKM for a proper comparison, especially for those analysis related to expression level quantification.

Response: Thanks for all of your valuable comments. According to your new suggestions, we calculated the FPKM of genes gained in the reference-free method and redid all subsequent reference-free analyses with FPKM and all comparisons related to expression level quantification. For these newly added parts, we have revised as follows:

“We also calculated the FPKM of each unigene (genes spliced in the reference-free research) to represent gene expression level instead of Reads Per Kilobase per Million mapped reads (RPKM) used in the research [22].” [lines 121-124, page 7]

“DEGs obtained in the reference-based method and the reference-free method were recorded separately.” [lines 133-135, page 8]

“We performed weighted correlation network analysis (WGCNA) using gene expression data obtained by two methods respectively to identify DEGs obtained by pairwise comparisons associated with acoustic resting frequency (RF) [31]. [lines 150-152, page 8]

And we also revised all changed results in the “Result” and “Discussion” sections.

Minor concerns/suggestions:

page 4 line 59: maybe deleting "splice" at this line, otherwise it doesn't make much sense.

Response: We have deleted the “splice” word. [line 59, page 4]

page 5 line 77: "basic" => "generic"

Response: We had changed “basic” to “generic”. [line 75, page 5]

page 6 line 100-108: Please provide the URL (or accession nu mber) for the reference genome/transcriptome/annotation used.

Response: Based on your suggestion, we had moved the URL of reference genome and annotation gene model files from lines 113 to 106. [lines 106-107, page 6]

page 11 line 192-196: Please state explicitly that the presented results in this paragraph are based on reference-based method.

Response: Since we redid all reference-free analyses, we had added the new reference-free results.

This part was revised to “We obtained a total of 4452 DEGs in the reference-based method, including 3579, 1308, and 1012 DEGs in the comparisons HN vs. JL, HN vs. YN, and YN vs. JL comparisons, respectively (S8 Table), and a total of 18003 DEGs in the reference-free method, including 15484, 2519, and 7468 DEGs in these three comparisons (S9 Table). Both the two methods showed that the HN vs. JL comparison had the most DEGs. Gene expression patterns of HN were more similar to those of YN than JL according to the hierarchical clustering heatmap (Fig. 1). But different results of the most same pair were gained using the two methods.” [lines 197-205, page 12]

page 12 line 202: "more annotated DEGs" => "more functionally annotated DEGs" . Same correction applies to Fig2 as well.

Response: We had changed “more annotated DEGs” to “more functionally annotated DEGs”. [lines 209, page 12 and lines 226, page 13]

page 14 line 244: Better to give a formal definition for "unigenes" here before referring to it.

Response: Based on your suggestion, we changed it to “Additionally, although almost all genes spliced in the reference-free method, which were called unigenes, were successfully matched to the reference genome…” [lines 246-247, page 14]

page 15 line 272: "the other" => "other"

Response: We had changed “the other” to “other”. [line 274, page 15]

page 18: To show the GO term and KEGG pathways differed between the reference-based and reference-free methods, maybe the authors could consider using parallel set plots (https://ggforce.data-imaginist.com/reference/geom_parallel_sets.html) for visualization.

Response: Based on your suggestion, we changed Fig. 4 to a Sankey diagram. Since there were too many GO terms, we only put KEGG plot in the main body and colored all rectangle shape. [lines 332-336, page 18]

GO result were put in the S4 Figure, and we only colored the terms shared by the two methods.

More detailed information of those GO terms and KEGG pathways were put in the supporting files Tables S17-S20.

page 20: "more genes were found": "more" relative to what? Please clarify the comparison intended here.

Response: To make the sentence clearer, we have changed this part to “Moreover, other key genes were found to be crucial to auditory phenotype differentiation in addition to DEGs…” [lines 375-376, page 20]

Reviewer #2: (No Response)

Response: Thanks for your recognition of our research.
